# Exacerbated metastatic disease in a mouse mammary tumor model following latent gammaherpesvirus infection

**DOI:** 10.1186/1750-9378-7-11

**Published:** 2012-05-29

**Authors:** Vinita S Chauhan, Daniel A Nelson, Lopamudra Das Roy, Pinku Mukherjee, Kenneth L Bost

**Affiliations:** 1 Department of Biology, University of North Carolina at Charlotte, 9201 University City Blvd, Charlotte, North Carolina, USA

**Keywords:** Breast cancer, Mammary tumor, Metastasis, Gammaherpesvirus

## Abstract

**Background:**

Controversy exists as to the ability of human gammaherpesviruses to cause or exacerbate breast cancer disease in patients. The difficulty in conducting definitive human studies can be overcome by investigating developing breast cancer in a mouse model. In this study, we utilized mice latently infected with murine gammaherpesvirus 68 (HV-68) to question whether such a viral burden could exacerbate metastatic breast cancer disease using a mouse mammary tumor model.

**Results:**

Mice latently infected with HV-68 had a similar primary tumor burden, but much greater metastatic disease, when compared to mock treated mice given the transplantable tumor, 4 T1. This was true for lung lesions, as well as secondary tumor masses. Increased expression of pan-cytokeratin and VEGF-A in tumors from HV-68 infected mice was consistent with increased metastatic disease in these animals. Surprisingly, no viral particles could be cultured from tumor tissues, and the presence of viral DNA or RNA transcripts could not be detected in primary or secondary tumor tissues.

**Conclusions:**

Latent HV-68 infection had no significant effect on the size of primary 4 T1 mammary tumors, but exacerbated the number of metastatic lung lesions and secondary tumors when compared to mock treated mice. Increased expression of the tumor marker, pan-cytokeratin, and VEGF-A in tumors of mice harboring latent virus was consistent with an exacerbated metastatic disease. Mechanisms responsible for this exacerbation are indirect, since no virus could be detected in cancerous tissues.

## Background

An association between Epstein Barr Virus (EBV) [1] and breast cancer continues to be controversial [2-5]. While EBV is clearly an etiologic agent in epithelial cell cancers like nasopharyngeal carcinoma and some gastric carcinomas [6-8], its importance in breast cancers remains uncertain. For more than 15 years, [9], numerous laboratory studies have reported the detection of EBV genomes, EBV RNA, or viral protein in breast biopsy tissues or cells [9-14]. The overriding notion for these studies was that if EBV was detected, even in a percentage of patients or even in a small percentage of breast cancer cells, that a possible etiology might be indicated. Alternatively, if EBV was not detected, this would eliminate this virus from consideration as a causative agent in breast cancers. If fact, other investigators have not been able to detect the presence of EBV in biopsied breast cancer tissues [15-20]. Thus, while it is difficult to prove such negative results due to possible technical limitations, the lack of positive results in some studies adds to the controversy. Even if detection methods become more sophisticated, more sensitive, and/or more reliable, the ability to detect EBV within human breast cancer tissue or cells would only suggest a role for this virus in some patients due to the ethical limitations of performing more definitive studies.

Less consideration has been given to the possibility that the presence of systemic EBV in some breast cancer patients might indirectly exacerbate disease without being present within the tumor cells themselves. Such a possibility has been suggested by studies which have correlated the presence of EBV in infiltrating lymphocytes [21], or where EBV reactivation was associated with pregnancy [22]. Furthermore, such a suggestion is not without precedence in disease states, since EBV-exacerbated autoimmune disease has been investigated for many years as an indirect result of viral infection [23-25].

Unfortunately, due to the limitations in patient studies, cause-effect relationships will remain difficult to prove. Whether the presence of EBV has a direct contribution, an indirect contribution, or no contribution to the etiology, exacerbation, or increased metastasis of some breast cancers will be difficult to define.

In the present study, we utilized an excellent mouse model of gammaherpesvirus infection to directly address the possibility that prior infection with an EBV-like gammaherpesvirus exacerbates metastases in mouse models of breast cancer. Murine gammaherpesvirus 68 (HV-68) mimics the pathophysiology of EBV [26,27], and has been used as a rodent model to investigate the host-pathogen interaction [28-33]. Upon intranasal or oral inoculation in mice [32], there is a productive infection of epithelial cells, followed by infection of B lymphocytes, and also macrophages and dendritic cells [26]. A marked leukocytosis (i.e. mononucleosis) and splenomegaly occurs, which peaks around 15 days post-infection and results in the establishment of latency for the life of the host. Given an appropriate stimulus [34], gammaherpesviruses can emerge from latency, resulting in a productive infection and the re-establishment of latency. The pathophysiology of murine gammaherpesvirus 68 closely mimics that observed for EBV infections [26,27], making this model a useful one for investigating such viral infections.

Similarly the syngeneic, transplantable mouse mammary tumor, 4 T1, which was utilized here, is a tractable model for the study of human breast cancers [35]. In the present study, we question whether mice latently infected with HV-68 had increased disease when compared to mock treatment. There were two surprising findings from this work. The size of primary mammary tumors did not differ between these two groups, however a significant increase in metastatic disease was observed in mice harboring this latent virus. Furthermore, it is highly unlikely that this exacerbated metastatic disease is the direct result of viral infection of 4 T1 cells since no infectious virus or viral RNA transcripts could be detected in tumor tissues.

## Results

### Morbidity of HV-68 infected mice as 4 T1 breast tumors develop

**Figure 1:**
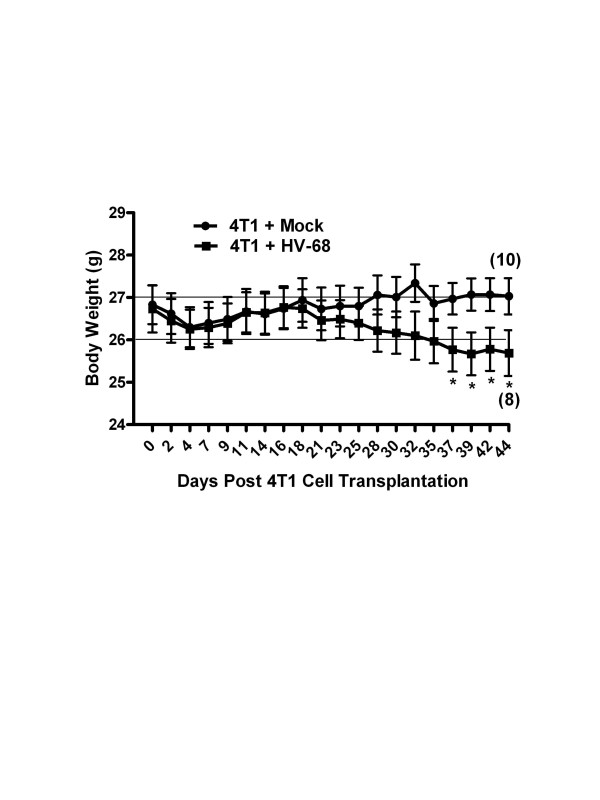
**Morbidity of HV-68 infected mice as 4 T1 breast tumors develop.** Groups of mice (N = 10) were mock treated (circles) or HV-68 infected (squares). Six months later mice were injected with syngeneic 4 T1 mammary tumor cells in the mammary fat pad. Mice were weighed and scored for morbidity following cancer cell transplantation. Results are shown as mean values (± standard deviations) with asterisks indicating a statistically significant difference between groups. Numbers in parentheses indicate the animals that remained alive when the experiment was terminated. This study was repeated twice with similar results.

To assess any clinical effects that gammaherpesvirus infection might have on developing breast cancer, we employed a mouse model of HV-68 infection [26-28] and the transplantable mammary tumor, 4 T1 [35]. Groups of mice were mock treated or infected with HV-68. Six months later after the virus had established latency, 4 T1 mammary tumor cells were injected into mammary fat pads of mice. The weight and general health of the animals were recorded as the mammary tumors developed. Approximately three weeks following mammary cell transplantation, there was a noticeable difference in the health of mice that were latently infected with HV-68 compared to the mock treated group. As shown in Figure 1, weight loss was accelerated in mice with a viral burden, and this experiment was terminated 44 days following transplantation when two of ten HV-68 infected mice succumbed to metastatic disease.

### Primary mammary tumor burden in mock and HV-68 infected mice

**Figure 2:**
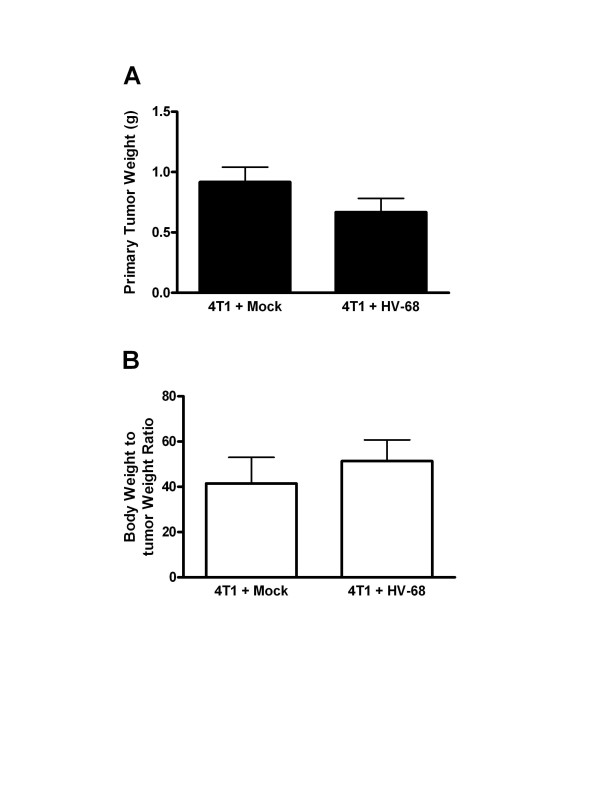
**Primary mammary tumor burden in mock and HV-68 infected mice.** At death or at day 44 following tumor cell transplantation, groups of mice (N = 10) were euthanized and primary mammary tumor tissue excised and weighed. Panel **A** shows mean tumor weights (± standard deviations). Panel **B** shows the ratio of average body weight to average tumor weight (± standard deviations) as a measure of tumor burden.

Mice were euthanized at 44 days following tumor cell transplantation to quantify mammary tumor burden in each animal. Figure 2A shows the average weights of mammary tumors in mock versus HV-68 infected mice. Since the body weights of mice in each group differed significantly by day 44 (Figure 1), it was important to factor in this difference when comparing tumor burden (Figure 2B). Regardless of the method of analysis, we could detect no significant difference in primary mammary tumor burden in mock versus HV-68 infected mice.

### Metastatic tumor burden in mock and HV-68 infected mice

**Figure 3:**
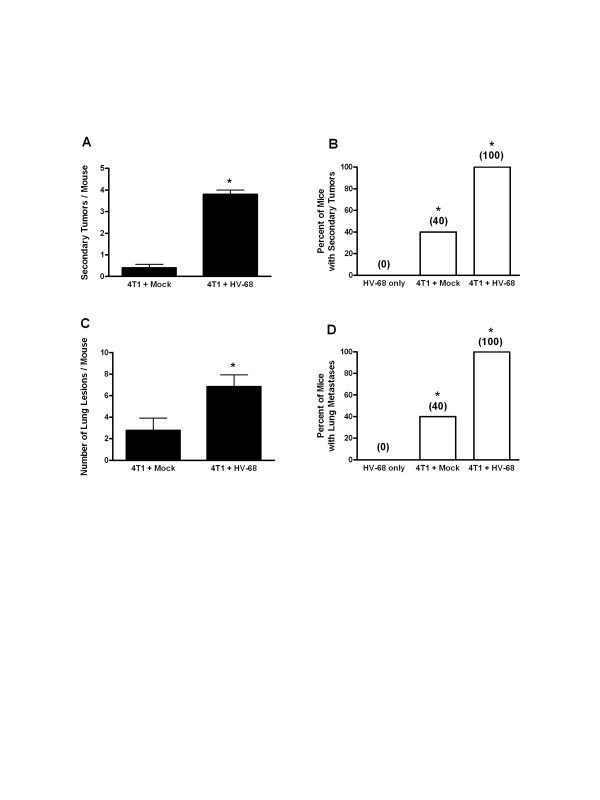
**Metastatic tumor burden in mock and HV-68 infected mice.** At day 44 following tumor cell transplantation, groups of mice (N = 8–10) were euthanized. Secondary tumors, lungs, and blood were taken from each animal. Panels **A** and **B**, respectively, show the average number of secondary tumors and percentage of mice with secondary tumors for each group (± standard deviations). Panel **C** and **D**, respectively, show the average number of lung metastases and percentage of mice with lung metastases for each group (+ standard deviations). Asterisks indicate statistically significant differences between groups.

Following euthanasia, metastatic tumor burden in peripheral organs and the lungs was also assessed. When laparotomies were performed, it was obvious that HV-68 infected mice had increased numbers of secondary tumors. Figure 3 shows a representative example of the average number of tumors per mouse in each group, with 100% of HV-68 infected mice having multiple metastases (Figure 3B). Similarly, lung lesions due to infiltrating tumor cells were significantly increased in HV-68 infected mice (Figure 3C), with 100% of mice harboring latent virus having multiple lesions (Figure 3D).

**Figure 4:**
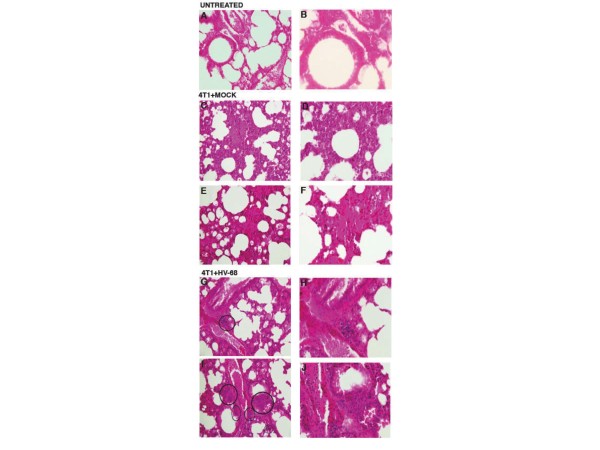
**H & E staining to visualize metastatic lung lesions in untreated, mock and HV-68 infected mice.** At day 44 following tumor cell transplantation, groups of mice (N = 8–10) were euthanized. Lungs were taken from each animal, fixed in formalin, and paraffin embedded for H & E staining. Left panels ( **A**, **C**, **E**, **G**, and **I**) show 20X magnification and right panels ( **B**, **D**, **F**, **H**, and **J**) show 40X magnifications of the same micrographs. Panels A and B show representative microscopic lung sections from an untreated mouse. Panels **C**, **D**, **E** and **F** show representative microscopic lung sections from two mock treated mice that were transplanted with 4 T1 tumor cells. Panels **G**, **H**, **I** and **J** show representative microscopic lung sections from two HV-68 infected mice transplanted with 4 T1 tumor cells. Circles show the location of clearly discernable metastatic lesions in HV-68 infected mice.

Representative histological sections are shown in Figure 4 as evidence of the differences between lung lesions in mock (Figures 4C and 4E) and infected mice (Figures 4G, and 4I). In HV-68 infected mice, areas of tumor cell accumulation were readily observed (circled regions in Figures 4G and 4I). Higher magnifications of these representative histological sections for mock treated (Figures 4D and 4F) and infected mice (Figures 4H and 4J) show similar differences in infiltrating tumor cells.

We concluded that the metastatic tumor burden in HV-68 infected mice was significantly increased when compared to mock treated animals (Figures 3 and 4). These observations were consistent with the increased weight loss and increased morbidity observed in latently infected animals (Figure 1).

### Pan-cytokeratin staining of lung and secondary tumor sections

**Figure 5:**
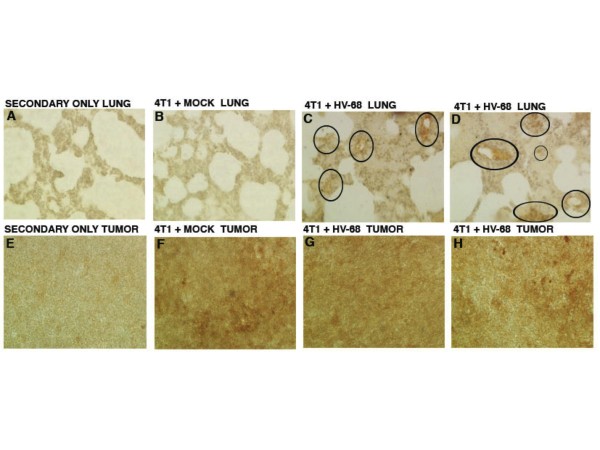
**Pan-cytokeratin staining of lung and secondary tumor sections.** At day 44 following tumor cell transplantation, groups of mice (N = 8–10) were euthanized. Lungs and primary tumor masses were excised from the animals. Tissue was fixed in formalin, paraffin embedded, and sectioned for staining with an antibody against pan-cytokeratin as a marker for 4 T1 cells. The chromogen, DAB, stains brown and was used to detect the presence of anti-pan-cytokeratin antibody binding to tumor cells. Panel B shows a representative anti-pan-cytokeratin stained microscopic lung section from a mouse transplanted with 4 T1 tumor cells. Panels **C** and **D** show representative anti-pan-cytokeratin stained microscopic lung sections from two HV-68 infected mice transplanted with 4 T1 tumor cells. Circled regions in Panels **C** and **D** indicate areas of increased staining for cytokeratins in infected mice. Pan-cytokeratin staining was also used to identify secondary metastatic tumor masses as being composed of 4 T1 cells. Tumor sections from a representative mock treated mouse (Panel **F**) and from two representative HV-68 infected mice (Panels **G** and **H**) showed similar staining for this tumor marker. It should be noted that due to increased mucus in lung tissues of tumor bearing mice, there was higher background staining as evident by the secondary antibody-only control (Figure 5A). This increased background staining was not observed in the control for primary tumors (Figure 5E).

4 T1 cells are epithelial in origin and express high levels of cytokeratins, allowing these proteins to mark the presence of infiltrating tumor cells. Therefore these proteins were used as markers for the presence of infiltrating cancer cells in lung and tumor tissues. To visualize 4 T1 tumor metastases in HV-68 infected mice, sections of lung and primary tumors were stained with an anti-pan-cytokeratin antibody. Lung sections from two representative HV-68 infected mice showed increased staining for cytokeratins (circled regions in Figures 5C and 5D), when compared to a lung section from a representative mock treated mouse (Figure 5B). These data are consistent with an increased metastasis of 4 T1 tumor cells into lung tissue of mice latently infected with this gammaherpesvirus.

We also utilized pan-cytokeratin staining to identify secondary metastatic tumor masses as being composed of 4 T1 cells. Tumor sections from a representative mock treated mouse (Figure 5F) and from two representative HV-68 infected mice (Figures 5G and 5H) showed similar staining for this tumor marker.

### Vascular Endothelial Growth Factor (VEGF-A) staining of primary mammary tumor sections

**Figure 6:**
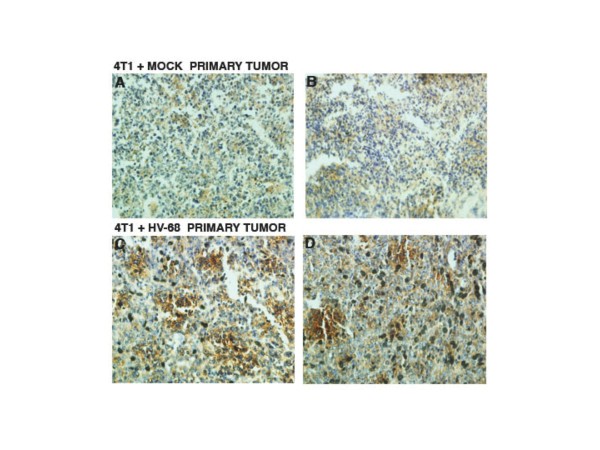
**VEGF-A staining of primary mammary tumor sections.** At death or at day 44 following tumor cell transplantation, groups of mice (N = 10) were euthanized. Primary mammary tumors were excised from each animal, fixed in formalin, paraffin embedded, and sectioned for staining with an antibody against VEGF-A as a marker for angiogenesis. The chromogen, DAB, stains brown and was used to detect the presence of anti-VEGF-A antibody binding. Panels **A** and **B** show representative anti-VEGF-A stained microscopic mammary tumor sections from two mock treated mice transplanted with 4 T1 tumor cells. Panels **C** and **D** show representative anti-VEGF-A stained microscopic mammary tumor sections from two HV-68 infected mice transplanted with 4 T1 tumor cells.

Increased expression of VEGF-A within primary breast tumors correlates with increased angiogenesis, as well as increased metastasis to secondary sites [36,37]. Based on the results in Figures 3,4, and 5, we anticipated that mice harboring latent virus would have increased VEGF-A expression in their primary tumors. Figure 6 shows representative primary tumor sections from two different HV-68 infected mice (Figures 6C and 6D) or from mock treated animals (Figures 6A and 6B). Increased intensity of VEGF-A staining can be seen in these sections (Figures 6C and 6D), which is consistent with an increased potential for tumor cell metastasis in HV-68 infected mice.

### No detectable HV-68 RNA transcripts in primary mammary tumors or secondary metastases

**Figure 7:**
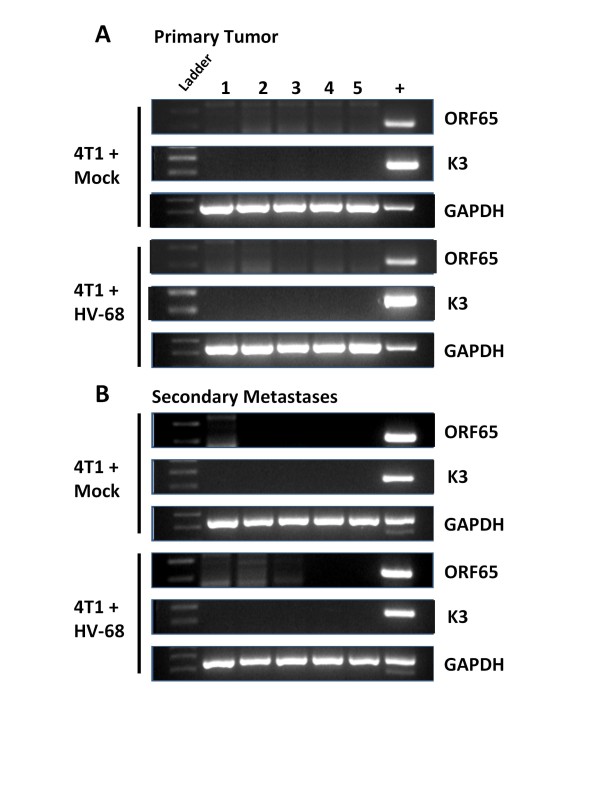
**No detectable HV-68 RNA transcripts in primary mammary tumors and secondary metastases.** At death, or at day 44 following tumor cell transplantation, groups of mice (N = 10) were euthanized. Primary mammary tumors and secondary metastases were excised from each animal, and RNA extracted from each tissue. Quantitative RT-PCR was performed to detect the presence of one replicating HV-68 transcript (ORF65) and one latent HV-68 transcript (K3). Results from 5 different animals (1, 2, 3, 4, 5) are shown as amplified fragments electrophoresed on ethidium bromide stained polyacryamide gels. The correct size of each amplified fragment is indicated by comparison to DNA ladders run on the same gel (Ladder). Positive controls for the amplification of each gene were also included in each reaction (+). The presence of GAPDH RNA was used as a positive control in each RT-PCR reaction.

One possible explanation for increased metastasis would be the presence of gammaherpesvirus in 4 T1 cells, as has been suggested for some EBV-associated breast cancers [9-14]. To address this possibility, we attempted to culture lytic or latent virus from tumor tissue using methods routine in our laboratories [29,30,38], with no success (data not shown). A more sensitive, and less technically demanding, technique was then used to detect viral transcripts. This very sensitive RT-PCR analysis [29,39,40] was used to detect the presence of any viral RNAs in primary mammary tumors and in secondary metastases that might be present. Two separate HV-68 RNAs were selected for analysis, including an RNA species expressed by replicating virus (ORF65), and one expressed in cells harboring latent virus (K3). Neither viral transcript could be detected in primary or secondary tumors (Figure 7). We were also unable to detect viral DNA using a sensitive PCR technique (data not shown) [29,39,40]. Together, these results make it highly unlikely that the increased metastases observed in HV-68 infected mice following 4 T1 transplantation is due to a viral infection of these tumor cells.

## Discussion

Greater than 90% of all individuals have been exposed to EBV [1], and this exposure usually occurs relatively early in life before the development of most breast cancers. Based on this fact, it might be logical to conclude that EBV could never cause or exacerbate breast cancers since almost all patients with these tumors have been previously exposed to the virus. The error in this logic lies in the fact that EBV infections, and the accompanying viral burden, can vary dramatically between individuals [24,41-45]. It has been suggested that more recent and sensitive methods of EBV quantification need to be utilized as a prognostic tool for EBV-associated diseases [41,42,44]. Stated simply, even though most individuals have been exposed to EBV, each person can have very different latent and replicating viral burdens. Because of this difference, it has been postulated that some individuals will demonstrate significant immune dysregulation in their struggle to keep such latent viral infections under control [46-48]. If true, it is easy to imagine how EBV might be capable of exacerbating diseases in some individuals and not others. Such a relationship has been suggested for the autoimmune diseases, multiple sclerosis [23,24], systemic lupus erythematosus [24,49], and rheumatoid arthritis [24,50], among others.

Investigating the relationship between EBV infection and breast cancer has been the subject of numerous investigations [2-5]. Some studies have demonstrated the presence of EBV genes or proteins in breast cancer biopsies [9-14], while other studies have not detected the virus [15-20]. One criticism of studies that have detected EBV genes is the low level of such expression, which questions the small percentage of tumor cells which might harbor virus and whether virus in non-tumor cells can be excluded. Some studies have correlated antibody reactivity against EBV antigens with the development of breast cancers, suggesting a recent or ongoing immune response against this viral infection. Other studies have suggested that there is a relationship between a subset of very aggressive breast cancers and the presence of EBV [51]. Whether the presence of this virus has a direct contribution, an indirect contribution, or no contribution to the etiology of breast cancers in a subset of patients remains unclear [2-5].

Here we investigated the possibility that the presence of a latent gammaherpesvirus might exacerbate disease in a transplantable breast cancer using mouse models. Combining HV-68 latency [26-28] with mice developing 4 T1 mammary tumors [35] allowed a direct analysis of whether this gammaherpesvirus could infect breast carcinoma cells in vivo, and whether disease was exacerbated when compared to uninfected animals. While primary tumor growth did not vary between these groups (Figure 2A and 2B), the health of mice infected with HV-68 began to decline dramatically during days 30 to 44 post 4 T1 transplantation (Figure 1). When HV-68 infected mice began to succumb at day 44 (Figure 1), the remaining mice were euthanized for analyses. It then became apparent why HV-68 infected mice were losing weight and were becoming moribund as metastatic lesions in the lungs and secondary tumors were accumulating in these animals (Figures 3 and 4). Compared to uninfected mice, we concluded that harboring latent HV-68 resulted in an exacerbation of metastatic disease during developing 4 T1 mammary tumors. Increased expression of pan-cytokeratin (Figure 5) and VEGF-A (Figure 6) was consistent with this conclusion.

The availability of sensitive assays to detect lytic and latent HV-68 allowed us to question whether primary or secondary tumor tissue contained virus. Tissue homogenates from tumors did not harbor latent or infectious viral particles (data not shown) when cultured on permissive cell lines [29,30,38]. Neither the presence of a representative lytic transcript (ORF 65) nor a representative latent transcript (K3) could be detected in any tumor tissue (Figure 7). This RT-PCR assay can detect less than one viral transcript in one microgram of total RNA [29,39,40]. In addition, viral DNA could not be detected (data not shown). Therefore it is highly unlikely that HV-68 or HV-68 infected cells are present in tumor tissues at a level that could explain the increased metastatic disease that was observed. These results do not support the notion that EBV exists within breast cancer cells themselves as a mechanism for exacerbated disease.

The most likely explanation for HV-68 exacerbated metastatic disease is an indirect one. Gammaherpesviruses, like EBV and HV-68, establish latency in B lymphocytes and other antigen presenting cells [1,26]. Reactivation from latency permits lytic virus to infect a variety of cells while stimulating an immune response that limits viral replication in immunocompetent individuals [1,26]. Whether factors expressed by virally infected cells, or the immune response that controls viral replication, or the immune dysregulation that permits the virus to persist, are responsible for HV-68 induced metastases is not presently clear. Such indirect mechanisms for EBV-exacerbated breast cancer disease in patients have not been investigated. Unfortunately, such definitive studies will be difficult to perform in patients. However the present studies suggest a useful model for investigating mechanisms responsible for gammaherpesvirus-exacerbated cancer.

One possible outcome for demonstrating gammaherpesvirus-exacerbated disease is the notion that anti-viral therapies would then be one co-treatment option for some breast cancer patients. Surprisingly, there is not much compelling data to suggest the presence of replicating EBV in studies using breast cancer biopsies [2-5]. Most viral RNAs and proteins which have been detected represent those expressed by latent virus. If replicative virus is not present during the course of developing breast cancer, most anti-viral therapies would not be effective [52]. Furthermore, there have been no studies to address the ability of developing breast cancer to induce EBV reactivation from latency in vivo. Using this mouse model of HV-68 infection, it will be possible to directly address such questions. In particular, it will be important to determine whether developing breast cancer can induce gammaherpesvirus reactivation and at what times during metastatic disease that replicative virus can be detected. If such future studies demonstrate viral reactivation during tumorigenesis, then the possibility that anti-herpesvirus therapies might limit virus-induced exacerbation of transplantable or spontaneous breast cancers could also be explored. Finally, the mechanisms responsible for HV-68 induced exacerbation of metastatic disease must be defined. While such studies would be difficult to perform in patients, the ability to use HV-68 as a model of gammaherpesvirus latency and reactivation will allow definitive studies to be performed.

## Methods

### Animals

Six to eight week old female BALB/c mice (18–22 g) were purchased from Jackson Laboratories (Bar Harbor, ME) and housed in the vivarium in filter top cages containing sterile bedding. After arrival, mice were quarantined for at least five days, and fed chow and water *ad libitum*. All animal experiments were in compliance with protocols approved by the University of North Carolina at Charlotte Animal Care and Use Committee.

### Murine gammaherpesvirus-68 (HV-68)

#### Maintenance of viral stocks

Murine gammaherpesvirus-68 (HV-68; ATCC # VR-1465) stocks were prepared by infecting baby hamster kidney cells (BHK-21; ATCC # CCL-10) at a low multiplicity of infection (MOI), followed by preparation of cellular lysates, as described previously [29,30,32].

#### Infection of animals

Groups of mice were anesthetized with isoflurane and mock treated by intranasal instillation of saline, or infected intranasally with 6 x 10^4^ plaque forming units of HV-68. At autopsy, animals were routinely screened for the presence of viral genomes to demonstrate that infection with virus had been successful in these animals (data not shown).

#### Assay of plaque-forming units in cell lysates

Plaque-forming HV-68 was quantified by adding 1:3 serial dilutions of BHK-21 cell lysates to BALB/3 T12-3 cell (ATCC # CCL-164) monolayers in 96-well plates. After the monolayers were incubated with virus for 1 hr, medium was removed and cells overlayed with 1% Plaque Assay Agarose (BD Biosciences, San Diego, CA) in medium with 30% fetal bovine serum. After 5–7 days in 5% CO_2_, overlays were removed and cell monolayers fixed for 1 hr with 4% formaldehyde and stained with 0.1% crystal violet. All serial dilutions were performed in triplicate.

### 4 T1 cells

4 T1 cells (highly metastatic; ATCC# CRL-2539) were used as model breast cancer cells [35]. Cells were cultured in ATCC complete growth medium (RPMI 1640 medium with 10% fetal bovine serum and 2 mM L-glutamine, adjusted to contain 1.5 g/L sodium bicarbonate, 4.5 g/L glucose, 10 mM HEPES, 1.0 mM sodium pyruvate and 0.05 mM 2-mercaptoethanol).

### Injection and monitoring of animals

Six months following mock treatment or HV-68 infection, mice with 4 T1 tumor cells. To produce tumors, 3.5x10^4^ 4 T1 cells in 50 ul of phosphate-buffered saline (PBS) were injected into the right abdominal mammary fat pad. Following injection, animals were monitored and weighed three times a week until the last week of the experiment, when they were monitored daily.

### Histology

Lungs and primary tumors were fixed in Prefer (Anatech, Battel Creek, MI) for a minimum of 24 hours post dissection. Paraffin embedded blocks were prepared and 8-micron thick sections were cut for histology and immune-staining. H&E staining was performed using a standard protocol [53] and pan-cytokeratin and VEGF-A staining was performed as previously described [35]. An anti-pancytokeratin antibody (Santa Cruz Biotechnology Inc., Santa Cruz, CA) and an anti-VEGF-A antibody (C-1, Santacruz Biotechnology Inc., Santa Cruz, CA) were used at a concentration of 1:50 overnight at 4^0^C. Dako anti-mouse IgG (Dako, North America) was used at a concentration of 1:100 for 45 minutes at room temperature. 3,3”- diaminobenzidine (DAB, Vector Laboratories, Burlingame, CA) was use as the chromagen and hematoxylin was used as a counterstain. Slides were visualized under light microscopy at 20X magnification.

### Detection of latent virus from tumor homogenates

Latent virus was quantified by adding 1:3 serial dilutions of homogenized tissue to BALB/3 T12-3 cell monolayers in 96-well plates. After the monolayers were incubated with virus for 24 hr, wells were overlayed with 1% Plaque Assay Agarose (BD Biosciences, San Diego, CA) in medium with 30% fetal bovine serum. After 5–7 days in 5% CO_2_, overlays were removed, cell monolayers fixed and stained with crystal violet. All serial dilutions were performed in triplicate.

### Nucleic acid analyses

#### Isolation of DNA

Five to twenty-five milligrams of tissue were suspended in 250 μl of ice-cold PBS and homogenized briefly in 1.5 ml microfuge tubes. An equal volume of PBS containing 2% SDS, 10 mM EDTA and 50 μg/ml Proteinase K (Sigma-Aldrich, St. Louis, MO) was added and homogenates incubated overnight at 37^o^C. Nucleic acid was extracted 2X with saturated phenol/chloroform/isoamyl alcohol (25:24:1), precipitated with 2 volumes of EtOH, and resuspended in PBS with 25 μg/ml RNase A (Sigma-Aldrich). After incubation for 30 min at 37^o^C, DNA was extracted 1X with phenol/chloroform/isoamyl alcohol, precipitated with 2 volumes EtOH, microfuged for 10 min at 16,000 x g and washed with 75% EtOH. The DNA pellet was air dried and resuspended in 10 mM Tris, pH 8.0. DNA concentration was determined by absorbance at 260 nm.

#### Preparation of cDNA

Total RNA was isolated using Trizol (Invitrogen; Carlsbad, CA), as previously described [29,54,55]. RNA samples were incubated with RNase-free pancreatic DNase (RQ1 DNase, Promega, Madison, WI) as per the manufacturer's instructions, the RNA precipitated with EtOH and resuspended in 50 μl of nuclease-free H_2_O. RNA concentrations were determined with a Gene Spec III spectrophotometer (Naka Instruments, Japan) using a 10 μl cuvette. For cDNA synthesis, one μg of RNA was reverse-transcribed in the presence of random hexamers (50 ng/μl), 10 mM dNTPs, 2.5 mM MgCl_2_ using ImProm-II reverse transcriptase (Promega) in the buffer supplied by the manufacturer. cDNA was precipitated with one-tenth volume of 3 M sodium acetate (pH 5.2) and 3 volumes of EtOH, and resuspended in 50 μl of nuclease-free H_2_O.

#### Semiquantitative PCR

Viral genomic DNA and mRNA transcript levels were examined by PCR. For semiquantitative PCR, 100 ng of DNA or cDNA was combined with 2.5 U of Taq polymerase (Promega), 0.2 mM each dNTP, 25 pmol of each primer and PCR buffer containing 2.5 mM MgCl_2_ as provided by the manufacturer. Samples were cycled using 95° denaturation for 35 seconds, 60°C annealing for 75 seconds and 72°C extension for 90 seconds, with the first three cycles using extended denaturation, annealing and extension times. PCR was for 35 cycles. The extension time of the last cycle was for 5 min at 72°C. Forty percent of each amplified PCR product was electrophoresed on an ethidium bromide-stained 2% agarose gel and photographed under UV illumination.

PCR primer sets were designed by using IDT SciTools and purchased from IDT (Integrated DNA Technologies, Coralville, IA). Primer sets used for amplification are as follows:

HV-68 ORF65 (open reading frame-65 - murid herpesvirus 4; accession no.NC_001826; 221 bp product):

Forward: 5' - ATG CTC CAG AAG AGG AAG GGA CAC - 3'

Reverse: 5' - TTG GCA AAG ACC CAG AAG AAG CC - 3'

HV-68 K3 (open reading frame-K3 - murid herpesvirus 4; accession no. NC_001826; 241 bp product):

Forward: 5' - TCT CAC GGG CTA ATC CAA GGT CAG - 3'

Reverse: 5' - GGG ACG TGG TTG CTG GTA AAT CAC - 3'

GAPDH (glyceraldehyde-3-phosphate dehydrogenase; accession no. NM_008084; 346 bp - exons 3 to 5):

Forward: 5' - CCA TCA CCA TCT TCC AGG AGC GAG - 3'

Reverse: 5' – CAC AGT CTT CTG GGT GGC AGT GAT - 3'

### Statistics

Data were analyzed using GraphPad Prism 5 software (GraphPad Software, Inc., San Diego, CA). Analyses were performed using Student's t-test, or by one-way analysis of variance (ANOVA) with Tukey’s Multiple Comparison Test as post-test. Mean values are presented in the figures +/− the Standard Error of the Mean (SEM). Results marked with an (*) were determined to be statistically significant at *P* < 0.05.

## Abbreviations

EBV: Epstein Barr Virus; HV-68: Murine gammaherpesvirus 68.

## Competing interests

The authors declare that they have no competing interests.

## Authors’ contributions

VSC, DAN, and KLB conceived and helped design the study and participated in data collection and analysis. LDR and PM helped design the study and participated in the analysis of data. All authors participated in writing and approving the final manuscript.
